# Species Composition, Natural Enemies, and Population Density of Pests in Greenhouse Banana Plantations of the Western Mediterranean Region of Türkiye

**DOI:** 10.3390/insects17020214

**Published:** 2026-02-18

**Authors:** Nurdan Topakcı

**Affiliations:** Department of Environmental Protection Technology, Vocational School of Technical Sciences, Akdeniz University, 07070 Antalya, Türkiye; ntopakci@akdeniz.edu.tr

**Keywords:** banana greenhouse, insect, mite, most abundance, parasitoid, pest population, predator

## Abstract

Banana (*Musa* spp.) is an economically important crop whose relevance is steadily increasing in greenhouse-based production systems. This study aimed to determine pest and natural enemy species, and pest population densities in greenhouse banana fields in the Western Mediterranean Region of Türkiye. Periodic studies were conducted every 15 days in 2.4 hectares for two consecutive years (2022–2023), while nonperiodic studies covered 128.9 hectares. Species were collected through visual inspection, sticky traps, and plant sampling. Seventeen pest species from four orders and eight families were recorded: *Frankliniella intonsa* (Trybom 1895), *Thrips hawaiiensis* (Morgan), *Thrips tabaci* Lindeman *Hercinothrips femoralis* (Reuter), *Pentalonia nigronervosa* Coquerel, *Rhopalosiphum maidis* (Fitch), *Brachycaudus helichrysi* (Kaltenbach), *Tetranychus turkestani* Ugarov & Nycolsky, *Tetranychus urticae* Koch, *Spodoptera littoralis* (Boisduval), *Bemisia tabaci* (Gennadius), *Aleyrodes* sp., *Planococcus citri* (Risso), *Dysmicoccus brevipes* (Cockerell), *Ceroplastes rusci* (L.), *Coccus hesperidum* L., and *Chrysomphalus aonidum* (L.). Twenty-two natural enemies from six orders and thirteen families were identified. This study also provides the first Turkish records of parasitoid *Coccophagus shillongensis* Hayat and Singh from *C. hesperidum* and *Encarsia aurantii* (Howard) from *C. aonidum*. The data showed that spider mites were the most abundant pests, while phytoseiid mites were their most abundant natural enemies. This study represents an important contribution to the scarce literature on insect and mite fauna associated with banana greenhouses in the Western Mediterranean Region. The findings are expected to contribute to the development of effective and sustainable pest management strategies for greenhouse banana production.

## Simple Summary

Banana (*Musa* spp.) is a globally important tropical fruit that can also be cultivated under subtropical conditions, with greenhouse production enabling year-round yields and improved fruit quality. It is an economically significant fruit that is increasingly cultivated in greenhouses, particularly in Türkiye. While greenhouse conditions enable year-round production, they also create favorable environments for insect and mite pests. Despite this, knowledge regarding pest and beneficial species associated with greenhouse banana cultivation in Türkiye remains limited. This study surveyed banana greenhouses in the Western Mediterranean Region during 2022–2023 to identify pest species, assess their population densities, and document their natural enemies. Seventeen pest species and twenty-two beneficial species were recorded. Spider mites represented the most prevalent pests, whereas predatory mites were the most abundant natural enemies. These findings provide valuable insights for the development of sustainable, integrated pest management strategies in greenhouse banana production.

## 1. Introduction

Banana (*Musa* spp.) is among the most important tropical fruit species cultivated worldwide for economic purposes [1]. Although primarily grown under tropical conditions, bananas can also be successfully cultivated in subtropical climates [2]. Greenhouse cultivation enables year-round production and significantly enhances both yield and fruit quality [3,4].

In greenhouse systems, the continuous availability of nutrients and the protected environmental conditions can lead to higher pest reproduction rates and more severe plant damage compared with open-field cultivation [5]. Greenhouses thus provide highly favorable habitats for pest development, making pest management a major component of production costs and a critical factor influencing both yield and crop quality [6]. The composition of pest species can vary markedly depending on geographic location and banana cultivar. Moreover, modifications in cultivation practices or the introduction of new or non-native cultivars may facilitate the emergence, establishment, or expansion of previously unrecorded pest species (7).

Existing research demonstrates that *Cosmopolites sordidus* (Germar 1824) (Coleoptera: Curculionidae) and plant-parasitic nematodes constitute the principal pest complex of certain banana locations [7]. Nevertheless, subsequent studies have revealed a broader assemblage of economically significant pests. Pinese and Piper [8] identified banana scab moth, thrips, spider mites, and the banana weevil borer as major pests in Queensland, Australia. Additional investigations have reported aphids, stem weevil, lace bug, stalk fly, whiteflies, banana weevil borer, Chinese rose beetle, mealybugs, rust thrips, rhizome weevils, fruit-scarring beetles, armored scales, and tingid bugs as pests across diverse banana-growing regions [9,10,11]. Mahalanobish et al. [12] also emphasized the significance of the fruit and leaf-scarring beetle as an important pest of banana. Chinese rose beetles and peer-scarring beetles were also mentioned [7]. To date, more than 470 insect and mite species have been documented to attack bananas at various phenological stages, the majority of which are foliar feeders (250), followed by fruit and flower feeders (130), root and rhizome feeders (70), pseudostem borers (10), and vectors of plant pathogens (10) [13].

Türkiye’s banana production—conducted predominantly under greenhouse conditions—has nearly doubled in recent years due to the expansion of production areas and an increasing number of growers [14]. In 2019, approximately 70% of Türkiye’s total banana yield was produced in greenhouses [15]. Despite this rapid growth, studies on banana pests in Türkiye remain limited. Existing research has focused primarily on plant-parasitic nematodes [16,17,18,19], while investigations on insect and mite pests are scarce. The few available studies on arthropod pests have reported only a small number of species, including *Sesamia nanogrioides* (Lefebvre 1827) (Lepidoptera: Noctuidae), *Cryptoblabes gnidiella* (Milliere 1867) (Lepidoptera: Pyralidae), and *Pentalonia nigronervosa* Coquerel 1859 (Hemiptera: Aphididae) [20,21,22].

The present study aimed to identify insect and mite species occurring on greenhouse-grown banana plants throughout the vegetation period, quantify the population densities of major pest species, and document beneficial species associated with these pests. The findings are expected to enhance knowledge of greenhouse banana cultivation and support the development of effective, evidence-based integrated pest management strategies.

## 2. Materials and Methods

### 2.1. Study Areas

**Figure 1:**
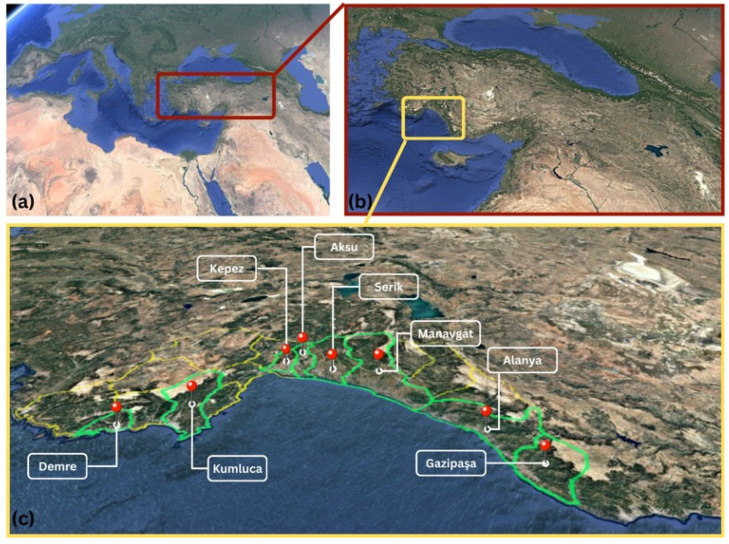
Türkiye (**a**), Western Mediterranean Region (**b**), greenhouse study areas (**c**). Source: Google Earth.

This study was conducted in greenhouse banana fields in the Western Mediterranean Region during 2022 and 2023 (Figure 1). Both periodic and nonperiodic field studies were performed to monitor pest population density throughout the year and to document species composition. In nonperiodic studies, only qualitatively results were provided. Periodic studies were carried out at 15-day intervals in three banana greenhouses totaling 2.4 hectares in the districts of Aksu, Serik, and Manavgat. Nonperiodic studies were conducted monthly in 71 selected greenhouses (different randomly selected greenhouses were visited each month) covering 128.9 hectares across eight districts with significant greenhouse banana-growing potential (Aksu, Alanya, Demre, Gazipaşa, Kepez, Kumluca, Manavgat, and Serik). The locations of the study areas are presented in Table 1. Standard agricultural practices (irrigation, fertilization, ventilation) were implemented as needed in all periodically and nonperiodically monitored sites. Depending on pest pressure from leafworms, spider mites, and aphids, pesticide applications were performed: Aksu (13 May 2022; 3 November 2022), Serik (5 November 2022), and Manavgat (5 June 2022; 28 July 2022; 21 November 2022; 7 June 2023). Additionally, dataloggers were installed in each periodically monitored greenhouse to record daily temperature and humidity values throughout the study period.

### 2.2. Sampling

In periodic visiting greenhouses, three methods were used to identify insect and mite species and their natural enemies on banana plants: visual examination, plant sampling, and sticky-trapping methods. In nonperiodic visiting greenhouses, only visual examinations were performed, and when necessary, plant samples were taken and examined macroscopically and microscopically in the laboratory.

#### 2.2.1. Visual Examination Method

Depending on the size of the sampling area, 30–40 randomly selected plants within each greenhouse were inspected by walking around each plant for 1–3 min. Leaves, stems, flowers, and fruits were examined visually to the naked eye or with a hand lens. Adult individuals detected during inspections were collected using a hand or mouth aspirator and placed into tubes containing 70% ethanol for successive identification. More active insects were evaluated directly on the plant. Sessile species were sampled together with the plant parts on which they occurred and transported to the laboratory in labeled plastic bags. The immature stages of pests and natural enemies were collected with infested plant material, first placed in paper bags, then transferred to polyethylene bags, and transported to the laboratory in an ice box. In the laboratory, the plant material with specimens was placed in glass jars covered with muslin cloth to allow the individuals to develop into adults. The emerged adults were preserved for species identification. This method allowed both mobile and immobile species, as well as their associated natural enemies, to be observed and recorded.

#### 2.2.2. Plant Sampling Method (Leaf, Flowers, and Fruits)

At 15-day intervals, a minimum of 30 plants were randomly selected in each greenhouse, and 30 leaf samples were taken from each plant. For noctuid species, whole leaves from selected plants were examined directly in the field. For other pests, approximately 20 cm^2^ leaf sections were taken from the middle or distal portions of the leaves. The collected samples were transported to the laboratory as described above. Leaf samples were examined under a stereo microscope. The total number of individuals was recorded to represent all species within each group. Larvae, nymphs, and adults of spider mites and phytoseiid mites; nymphs of whiteflies; nymph and adults of mealybugs; larvae and adults of thrips; nymphs and adults of aphids, larvae and pupae of noctuids, larvae and adults of coccinellids, and larvae and pupae of cecidomyiids were counted. During the plants’ flowering and fruiting periods, at least 10 flower and 10 fruit samples were examined in addition to the previous 30 leaf samples. Adult specimens found on both leaf surfaces were collected under a stereomicroscope with a fine brush and preserved in 70% ethyl alcohol. Immature stages were reared until they developed into adults as described above and stored for taxonomic identification. Almost all of the samples were sent to experts for identification.

#### 2.2.3. Trapping Method

To monitor pest populations, sticky traps were installed in the periodically monitored greenhouses on 22 January 2022 in the Aksu and Manavgat banana greenhouses, and on 8 February 2022 in the Serik greenhouse. One trap of each color was placed in each greenhouse, and these traps were replaced at 15-day intervals over a two-year period. Yellow traps used for monitoring whiteflies and aphids, and blue traps used for tracking thrips, were collected at each sampling event, placed between sheets of waxed paper, and transported to the laboratory. The pests captured on both surfaces of the traps were counted and recorded using a stereomicroscope. Natural enemy species captured in the traps were not evaluated; instead, these species were assessed solely through plant sampling and direct on-plant observations.

### 2.3. Identification of Specimens

The samples, labeled with collection location and date, were examined under a stereomicroscope and sorted to the family level. They were then sent to taxonomic experts for species-level identification. During field studies, both pest and beneficial species were photographed to create a digital archive.

## 3. Results

### 3.1. Species Composition of Pests and Natural Enemies in Banana Greenhouses

A total of 39 insect and mite species, representing 8 orders and 20 families, were identified in 74 banana greenhouses in the Western Mediterranean Region. The pest species are presented in Table 2, and the natural enemy species are provided in Table 3.

*Pentalonia nigronervosa* Coquerel 1859, the only aphid species identified during the periodic study, was first detected in Manavgat in the *initial* year. The pest was also recorded in numerous nonperiodic greenhouses, where it caused significant damage. *Brachycaudus helichrysi* (Kaltenbach 1843) was detected in a greenhouse on 2 March 2023 and in three different banana greenhouses on 21 March 2023 during nonperiodic surveys. *Rhopalosiphum maidis* (Fitch 1856) was detected in two separate banana greenhouses during nonperiodic sampling in March 2023. *Aphidius colemani* Viereck 1912 was identified as a parasitoid of the pest. Mealybug *Planococcus citri* (Risso 1813) was identified in both periodic and nonperiodic visiting greenhouses. Another mealybug species *Dysmicoccus brevipes* (Cockerell 1893) was recorded on plant roots during nonperiodic studies. The diaspidid *Chrysomphalus aonidum* (L. 1758), a member of Hemiptera, was recorded in two greenhouses in Alanya, while the coccid *Coccus hesperidum* L. 1758 was detected in a banana greenhouse in Kumluca during nonperiodic studies. *Bemisia tabaci* (Gennadius 1889) was widespread in both periodically and nonperiodically visited greenhouse areas. The thrips species recorded on banana plants were *Thrips hawaiiensis* (Morgan 1913)*, Frankliniella intonsa* (Trybom 1895)*, Thrips tabaci* Lindeman 1889, and *Hercinothrips femoralis* (Reuter 1891). Spider mites *Tetranychus turkestani* (Ugarov & Nycolsky 1937) and *Tetranychus urticae* Koch 1835 were recorded in nearly all greenhouses, in both periodic and nonperiodic studies. In both studies, *Spodoptera littoralis* (Boisduval 1833) is a commonly found species in banana greenhouses. Additionally, numerous oribatid mites were also detected on banana leaves. Although snails were found on banana leaves, stems, and fruits, they did not appear to cause direct feeding damage; however, the irregular white mucus trails they produced resulted in visible blemishes on plant surfaces.

**Figure 2:**
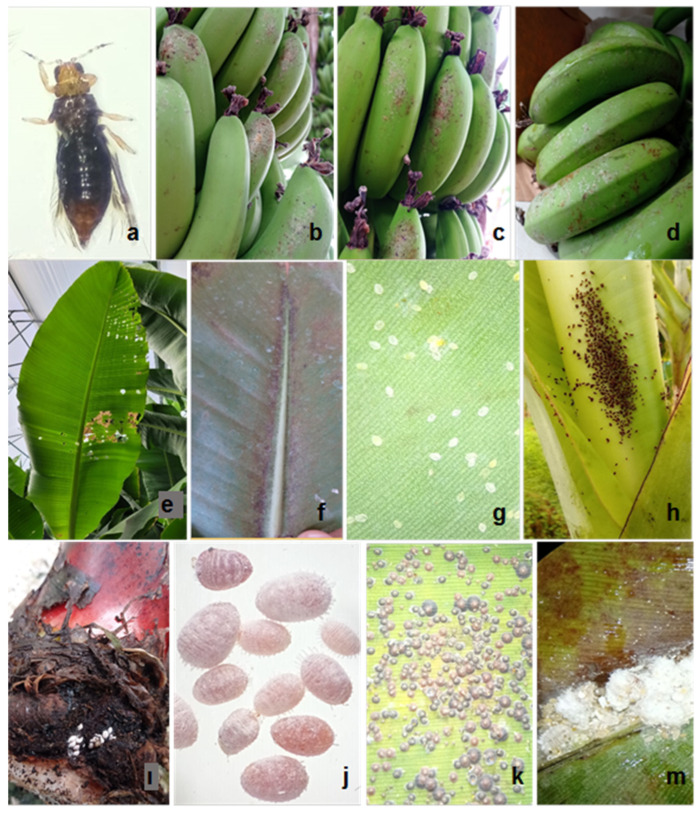
Adult of *Hercinothrips femoralis* (**a**), *Hercinothrips femoralis* damage on fruit (**b**–**d**), *Spodoptera littoralis* damage (**e**), *Tetranychus* spp. damage (**f**), *Bemisia tabaci* nymphs (**g**), nymph and adults of *Pentalonia nigronervosa* (**h**), nymph and adult of *Dysmicoccus brevipes* (**i**,**j**), adult and immature stages of *Chrysomphalus aonidum* (**k**), nymph and adult of *Planococus citri* (**m**).

**Figure 3:**
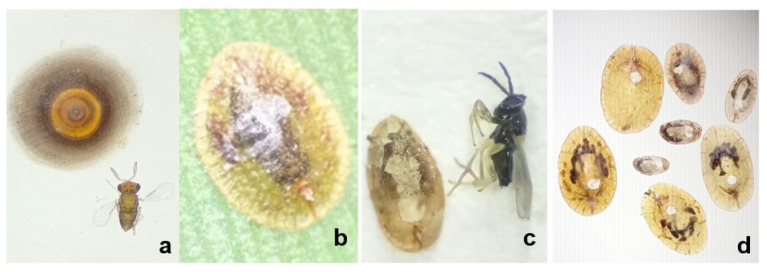
*Chrysomphalus aonidum* and its parasitoid *Encarsia aurantii* (**a**), *Coccus hesperidum* (**b**), adult and emergence holes of *Coccophagus shillongensis* on different biological stages of *Coccus hesperidum* (**c**,**d**).

Figure 2 shows images of the *H. femoralis*, *Tetranychus* spp., *B. tabaci*, *P. nigronervosa*, *D. brevipes*, *C. aonidum*, *P. citri*, and damage symptoms of *H. femoralis*, *S. littoralis*, and *Tetranychus* spp. observed in the study. Figure 3 shows the parasitoid *Encarsia aurantii* (Howard 1894) and *Coccophagus shillongensis* Hayat and Singh 1989.

### 3.2. Population Monitoring in Banana Greenhouses with Traps and Plant Sampling

**Figure 4:**
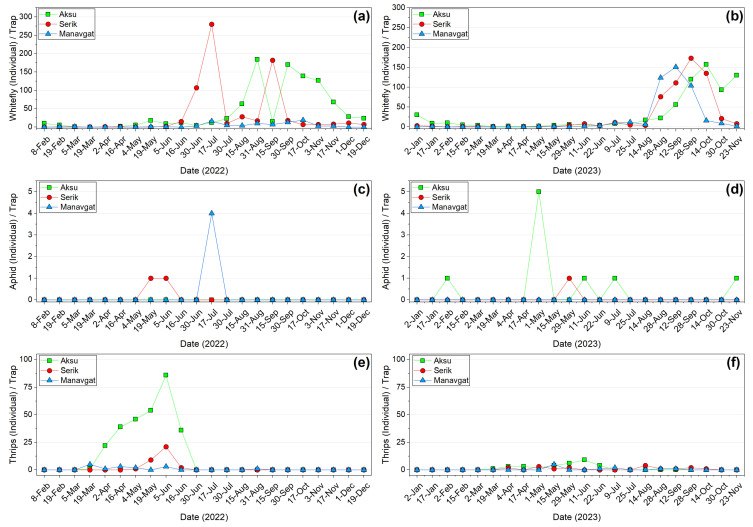
Pest density on traps in 2022–2023. Whitefly (**a**,**b**), aphid (**c**,**d**), and thrips (**e**,**f**).

The population densities of whiteflies, aphids, and thrips captured on yellow and blue sticky traps placed at 15-day intervals in banana greenhouses are presented in Figure 4. Whiteflies were consistently captured in greater numbers than the other pests. In the first year, the whitefly population in Serik began to increase from June, exhibiting two distinct peaks in July and September. In Aksu, whitefly numbers increased from August and, despite fluctuations, continued to be captured in traps until the end of the year. No pronounced population increase was observed in Manavgat during this period. In the second year, whitefly populations at all three locations increased from August. Although population levels declined after October, trap captures remained higher in Aksu compared with the other locations. The highest whitefly density was observed in Serik, with 280 individuals per trap in the first year and 173 individuals per trap in the second year. Aphid populations remained low at all sites throughout both years, with a maximum of 4 aphids per trap in Manavgat in July 2022 and 5 aphids per trap in Aksu in May 2023. In 2022, it was determined that thrips numbers gradually increased in both Aksu and Serik, peaking on 5 June, but at different intensities. Thrips were most abundant in Aksu, peaking at 86 individuals per trap in the first year and 9 individuals per trap on 11 June in the second year. The maximum thrips densities were 21 and 5 individuals per trap in Serik and Manavgat in the first year, respectively. In 2023, the number of thrips caught in traps did not exceed 5 in Serik and Manavgat.

**Figure 5:**
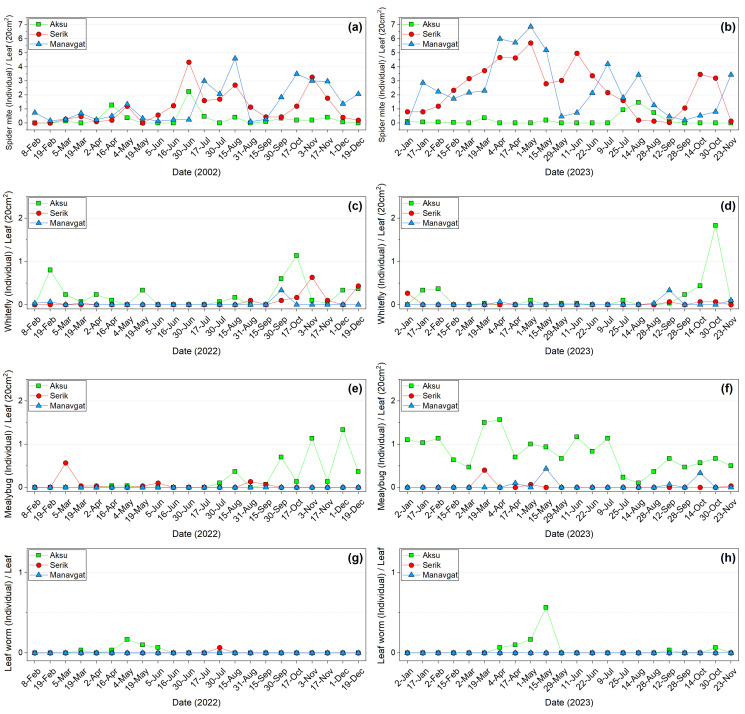
Pest density on leaves in 2022–2023. Spider mite (**a**,**b**), whitefly (**c**,**d**), mealybug (**e**,**f**), and leaf worm (**g**,**h**).

Pest densities obtained from plant sampling in periodically monitored banana greenhouses are presented in Figure 5. Spider mites were detected on banana leaves throughout nearly the entire season in all three greenhouses. The highest densities were recorded in Manavgat, with 4.60 individuals/leaf part on 15 August in the first year and 6.87 individuals/leaf part on 1 May in the second year. In Aksu and Serik, peak densities in the first year were observed on 30 June, reaching 2.23 and 4.33 individuals/leaf part, respectively. In the second year, the highest densities were 1.47 individuals/leaf part in Aksu on 14 August and 5.70 individuals/leaf part in Serik on 1 May. Spider mites peaked in Serik at the end of June and in Manavgat in mid-August in 2022, and again in both locations at the beginning of May in 2023, although their distribution fluctuated throughout both years. In Manavgat, however, these pests were recorded in lower numbers than in other locations. Whiteflies exhibited the highest densities in Aksu during both years, with 1.13 individuals/leaf part in the first year and 1.83 individuals/leaf part in the second year, both recorded in October. In Serik and Manavgat, whitefly densities did not exceed 0.6 individuals/leaf part. In Aksu, whitefly populations fluctuated at the beginning and middle of the season in both years. In contrast, fluctuations in Serik and Manavgat were less pronounced. Mealybugs were observed more frequently in Aksu than in the other locations, with peak densities of 1.33 individuals/leaf part on 1 December 2022 and 1.57 individuals/leaf part on 4 April 2023. In Serik, maximum mealybug densities were recorded in March in both years, at 0.57 and 0.40 individuals/leaf part, respectively. In Manavgat, mealybug densities did not exceed 0.4 individuals/leaf part. In the mealybug numbers recorded in Aksu, densities varied from July of the first year through to the end of the second year. In contrast, Serik and Manavgat exhibited more limited fluctuations, characterized by lower frequency and reduced population sizes. In Aksu, leafworm densities were observed on 4 May in the first year (0.17 larvae/leaf) and on 15 May in the second year (0.57 larvae/leaf). The pest was absent in Manavgat and was detected only once in Serik during the first year (July, 0.07 larvae/leaf). Aphids (*P. nigronervosa*) were observed only in the first year in Manavgat, with a peak of 0.27 individuals/leaf part on 8 February; none were detected in the second year. Fig wax scale was observed exclusively in Manavgat during the first year, reaching 0.37 individuals/leaf part on 16 June. Thrips were recorded in traps but not in leaf samples. No pests were observed on flowers or fruits in periodically monitored greenhouses. Thrips and aphid damage on banana fruits was detected in nonperiodic greenhouses.

**Figure 6:**
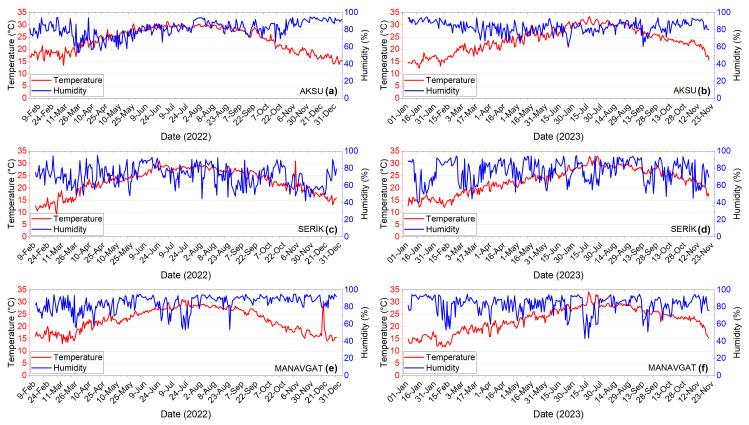
Temperature and relative humidity in periodic greenhouses in Aksu (**a**,**b**), Serik (**c**,**d**), and Manavgat (**e**,**f**) during 2022–2023.

In the periodically visited greenhouses, the daily average temperature and humidity values ranged from 12.3 to 33.3 °C and 53.4% to 95% in Aksu, from 9.5 to 33.0 °C and 40.8% to 95% in Serik, and from 11.7 to 34.1 °C and 43.4% to 95% in Manavgat (Figure 6).

Spider mites (Tetranychidae) were the most abundant pest species in periodically monitored banana greenhouses, followed by Pseudococcidae and Aleyrodidae. Their abundance in Serik and Manavgat exceeded 95% of all pest families noted (Table 4). The diversity and relative abundance of natural enemies associated with spider mites in periodically monitored greenhouses are presented in Table 5. Among these natural enemies, members of the family Phytoseiidae were the most abundant, accounting for 53.5% of the total. Thripidae constituted 27.8%, followed by Cecidomyiidae at 13.9%, and Coccinellidae at 5%.

## 4. Discussion

The study employed both periodic and nonperiodic methods. In both approaches, pest and beneficial species in banana greenhouses were identified, and pest population densities were monitored with periodic studies. During nonperiodic monitoring, only a qualitative assessment of the species present was made. While Mahanta et al. [23] reported that banana hosts comparatively fewer insect species, Shankar et al. [13] documented that many insect and mite species may attack bananas at different growth stages. In the present study, a total of 15 insect and 2 spider mite pest species and 22 natural enemy species were identified in greenhouse banana cultivation.

In this study, the mealybug *P. citri* was detected in leaf samples collected from routinely monitored greenhouses in both years, and it was also found on banana leaves in other surveyed greenhouses. Schmutterer and Cruz [24] reported that this species forms scattered colonies on banana pseudostems beneath the leaf sheaths. Karacaoğlu and Satar [25] noted that *P. citri* has a wide host range, including citrus and ornamental plants, and causes damage not only through feeding but also by inducing sooty mold development and acting as a virus vector. Another mealybug species identified in this study, *D. brevipes*, was observed in a nonperiodic sampling. Although the study focused on leaf, flower, and fruit sampling, *D. brevipes* was listed as a pest because it was detected on plant roots during greenhouse examination. *D. brevipes* has been reported as one of the most important invasive mealybug species worldwide [26]. It has also been reported to infest pineapple roots, leaves, fruits, blossom cups, and crowns [27]. In addition to being recorded on bananas in Hawaii and Uganda, the species has also been associated with Banana Streak Virus (BSV) [28,29].

The parasitoid *E. aurantii*, previously reported from 69 locations primarily in the Palearctic region [30], was identified in Türkiye for the first time in this study from *C. aonidum*. Similarly, *C. shillongensis* represents a new record for Türkiye. *C. shillongensis* was first described by Hayat and Singh in 1986 from an unidentified coccid species in India [31]. Outside India, the species was first detected and redescribed in Greece [32], followed by records from Serbia [33] and France [34]. In these few limited studies, *C. shillongensis* has been documented to parasitize 14 host species, including *C. hesperidum*, *Coccus pseudomagnoliarum* (*Kuwana* 1914) (Hemiptera: Coccidae), *Eulecanium tiliae* (Linnaeus 1758) (Hemiptera: Coccidae), *Parthenolecanium corni* (Bouché 1844) (Hemiptera: Coccidae), *Pulvinaria hydrangeae* (Stein. 1946) (Hemiptera: Coccidae), *Pulvinaria vitis* (*Linnaeus* 1758) (Hemiptera: Coccidae), *Aonidiella aurantii* (*Maskell* 1879) Hemiptera: Diaspididae), *Ceroplastes japonicus Green 1921* (Hemiptera: Coccidae), *C. rusci*, *Ceroplastes sinensis Del Guercio* 1900 (Hemiptera: Coccidae), *Filippia follicularis* (*Targioni Tozzetti 1867*) (Hemiptera: Coccidae), *Protopulvinaria pyriformis* (Cockerell 1894), (Hemiptera: Coccidae), *Pseudococcus viburni* (Signoret 1875) (Hemiptera: Pseudococcidae), and *Saissetia coffeae* (Walker 1852) (Hemiptera: Coccidae) [31,32,33,34]. The current study demonstrates that this parasitoid species parasitizes both adult and pre-adult stages of *C. hesperidum* (Figure 3c,d).

*P. nigronervosa* (banana aphid) is a widely distributed aphid species in tropical and subtropical regions, as well as in greenhouses in Europe and North America [35]. Although the pest primarily feeds on plants of the Musaceae family, there are also a few records on Heliconiaceae and Zingiberaceae [36]. It has been reported that the pest congregates with *Aphis gossypii* Glover, 1877 (Hemiptera: Aphididae) at the base of outer leaves on pseudostems and around the crown [13]. Hooks et al. [37] demonstrated that *P. nigronervosa* is more prevalent on young banana shoots than on mature plants, but also noted that mature plants have a relatively high potential for hosting this pest. Iesa [11] reported that banana aphid is the dominant aphid species in bananas and is present in all banana cultivation areas. Mahanta et al. [23] recorded this pest as being associated with banana in a horticultural orchard with a relative abundance of 2.64%, and identified it as a more abundant species than beetles and lepidopteran. In the present study, the abundance of banana aphid among other pest species was determined to be 1.4%. The banana aphid is a significant pest of banana and a vector of banana bunchy top virus [38,39]. *Banana bunchy top virus* (BBTV; Nanoviridae, Babuvirus) is one of the major diseases threatening production in a quarter of the world’s banana-growing areas [40]. *P. nigronervosa*, first reported in Türkiye in 2020, was identified as the only aphid species widespread in both open-field and greenhouse banana production areas, and no natural enemies were detected [22]. The current study demonstrates that the species has also spread to banana greenhouses in the Western Mediterranean region. Additionally, the parasitoids *A. colemani* and *P. abjectum*, as well as the predators *Scymnus levaillanti* (Mulsant 1850) and *P. quadrifasciatus*, were identified as natural enemies of this pest. Similarly, Poorani et al. [41] found that certain *Scymnus* species prey on the banana aphid. Other aphid species identified in the present study were *B. helichrysi* and *R. maidis*. *B. helichrysi*, widespread species that primarily feed on *Prunus* spp. (Rosaceae) with secondary hosts including Asteraceae, Boraginaceae, Fabaceae, and various ornamental plants [42]. The corn leaf aphid, *R. maidis*, is the most economically damaging aphid pest of maize (*Zea mays* L.) and also infests various monocot species [43]. Musaceae is listed among its hosts, including *Musa sapientum* L. [44]. *A. colemani* was identified as a parasitoid of *R. maidis*. Additionally, *A. vulgaris, A. citripes* (Thomson, 1862), and *Syphophagus* sp. have been recorded as hyperparasitoids of the parasitoid *A. colemani*.

Another hemipteran species identified in banana greenhouses is *B. tabaci*, the most frequently detected species in traps and observed in leaf samples throughout the season. Additionally, whiteflies belonging to the genus *Aleyrodes* were detected on young banana plants in Aksu. It was determined that these whiteflies migrated from weeds in the greenhouse to the banana plants, and reached larval and pupal stages but did not establish a significant population on the bananas. Therefore, it was not included in the whitefly counts. Furthermore, *Aleyrodes* sp. was also found to be heavily parasitized by *Eretmocerus mundus* Mercet 1931 on banana plants. Parasitized individuals, which were abundant in weeds and also found on banana leaves, were cultured in the laboratory to obtain adult parasitoids. No parasitized individuals were found on leaves where *B. tabaci* was present.

Thrips species were detected in sticky traps during periodic monitoring in all greenhouses, but were not found in leaf samples; however, they were determined on leaves during nonperiodic studies. *T. hawaiiensis*, commonly known as the flower or banana thrips, is a widespread polyphagous species in the Eastern and Pacific regions [45]. In Türkiye, it was first recorded on lemon in 2015 and rapidly spread in the Eastern Mediterranean Region, causing silvery spots and deformities on fruits [46]. This species oviposits during banana flowering and feeds during and shortly after flowering, resulting in raised silvery lesions and corky scarring on the fruit surface [45]. Other thrips species reported in banana fields in Australia, Central and South America, Oriental, and Pacific Regions include *Chaetanaphothrips orchidii* (*Moulton* 1907), *C. signipennis* (*Bagnall* 1914), *Frankliniella parvula* Hood 1925, *Heliothrips haemorrhoidalis (Bouché 1833*)*, Hercinothrips bicinctus* (Bagnall 1919), and *Tryphactothrips* sp. [45]. This study found that thrips species other than *H. femoralis* did not cause significant damage to bananas. In a few nonperiodic greenhouses, thrips species were found in low numbers, except for *H. femoralis*. In the greenhouse where *H. femoralis* was detected (1 hectares), approximately 0.5% of the plants (20 out of 4000) exhibited significant fruit damage associated with feeding and oviposition, resulting in discoloration (Figure 2a–d). The literature indicates that the first record of this pest in Türkiye was reported by Alkan in 1962, where it was included among pest species without supporting data [47]. The same study [47] reported that *H. femoralis* had not been monitored or reported again in Türkiye since that initial record. Originally described from Africa, *H. femoralis* has been reported from greenhouses in many subtropical and temperate regions. [48,49]. It was first recorded in Europe in Slovenia [50], followed by Greece [51], Croatia [52], and Slovakia [53]. In Korea, the species was first detected in 1973 and was recently rediscovered, causing significant damage to ornamental plants [54]. *H. femoralis* is polyphagous, with host records including figs, sugar beet, groundnuts, cotton, pineapple, sugarcane, ornamentals, maize, cabbage, and sweet potato [49,50,53,54,55,56]. It has also been documented on banana (*Musa* sp., Musaceae) in various locations [49,51]. Stefanik et al. [57] demonstrated that human passive transport plays a significant role in the spread of *H. femoralis.* The recent increase in reports from more countries highlights the potential for this species to cause damage and the need for increased attention [53]. The potential threat posed by *H. femoralis*, particularly in regions with intensive greenhouse production, warrants careful consideration.

Spider mites are a significant threat to agricultural production, infesting more than 1000 plant species, including various banana cultivars [58]. About 46 species of phytophagous mites from the family Tetranychidae have been documented on *Musa* spp. globally [59]. Greenhouse conditions facilitate the proliferation of spider mite populations, which are found on banana plants grown in these environments [60]. In the current study, high populations of spider mites caused discoloration and reddening of entire leaf surfaces, which were recorded. Topakcı et al. [61] previously identified *T. urticae* on banana leaves grown under greenhouse cultivation. *T. urticae* infestation is associated with a dull silvery appearance at the tips of banana fruit fingers, reduced photosynthetic capacity, and inhibited plant growth. Additional stress factors, such as drought or nematode infestation, further intensify the adverse effects [45]. Several mite species, including *Tetranychus lambi* Pritchard & Baker 1955, (Acari: Tetranychidae), *Tetranychus lombardinii* Baker & Pritchard 1960 (Acari: Tetranychidae), *Brevipalpus obovatus* Donnadieu 1875 (Acari: Tenuipalpidae), and *Calacarus citrifolii* Keifer 1955 (Acari: Eriophyidae), have been documented to cause seasonal damage to banana fruits [62]. *Tetranychus gloveri* Banks 1900 (Acari: Tetranychidae) has also been documented in several countries [63]. Escobar-Garcia and Andrade [64] identified *Tetranychus abacae* Baker & Pritchard 1962 (Acari: Tetranychidae) as the predominant mite species in banana, present in all 100 cm^2^ leaf samples across every sampling date. The highest densities of *T. abacae* were recorded at the beginning and middle of the study, with 15.12 and 8.27 mites per leaf, respectively [64]. Kar et al. [65] reported that the *Oligonychus sapienticolus* Gupta 1976 (Acari: Tetranychida e) was present on banana throughout the year, reaching a maximum density of 4.44 mites per 3 cm^2^. The maximum observed density of spider mites in periodically monitored greenhouses was 6.87 individuals per 20 cm^2^ of leaf samples.

In the present study, periodic leaf sample examinations revealed that spider mites were the most abundant pest species on banana plants. Among the predators of spider mites from the Phytoseidae, Thripidae, Coccinellidae, and Cecidomyiidae families, phytoseiids ((*Kampimodromus aberrans* (Oudemans 1930) and *Neoseiulus californicus* (McGregor 1954)) were the most prevalent (53.5%), followed by *Scolothrips longicornis* Priesner 1926 (27.8%). Dayoub et al. [66] found that on Solanaceous plants, *Feltiella acarisuga* (Vallot 1827) was the most common and abundant predatory insect among phytophagous mite predators, followed by *Stethorus gilvifrons* (Mulsant 1850) and *S. longicornis*. *Phytoseiulus persimilis* Athias-Henriot 1957 (Acari: Phytoseiidae) was identified as the most frequently encountered and abundant predatory mite. The present study found that the cecidomyiid predator *F. acarisuga* was parasitized by *Aphanogmus* sp. on banana leaves. Previous research in strawberry greenhouses demonstrated that the hyperparasitoid *Aphanogmus* sp. could parasitize *F. acarisuga* at rates of up to 50 percent [67]. Additional cecidomyiid species identified in this study belonged to the genus *Lestodiplosis.* Numerous *Lestodiplosis* species have been documented as specialist predators on various hosts, including mites, cecidomyiid larvae, lepidopterous caterpillars, and aphids [68,69].

Severe feeding damage caused by the noctuid *S. littoralis*, particularly during the early growth stages of banana plants, is attributed to its movement from weeds within the cultivation area. Therefore, targeted weed management is essential in newly established banana plantations to suppress *S. littoralis* infestations. Okkole et al. [70] similarly reported that lepidopterans are among the most destructive pests of banana foliage, with the mature larva of *Spodoptera litura* (Fabricius 1775) (Lepidoptera: Noctuidae) causing the greatest damage to young plants. *S. litura* prefers shorter plants to facilitate easier access to soil for pupation, resulting in higher infestation and damage levels one or two months after planting. In the Canary Islands, *Chrysodeixis chalcites* (Esper 1789) (Lepidoptera: Noctuidae) has been identified as one of the most significant pests in greenhouse banana production, with fruit losses increasing in recent years due to larval feeding [71].

Spiders (Araneae) represent another group identified in the present study. As abundant generalist predators, spiders help regulate populations of insect pests and disease vectors across diverse ecosystems [72] and contribute substantially to suppressing plant pests in agricultural systems [73]. In several banana greenhouses, high spider web densities were observed, occasionally hindering field activities.

In commercial greenhouse production, chemical control measures are often unavoidable due to increasing pest populations. Considering this, both periodically monitored greenhouses and randomly selected nonperiodic greenhouses were examined simultaneously for pest and beneficial species. The presence and distribution of species were assessed across greenhouses with different production conditions and pest management practices. This combined approach allowed for a more comprehensive assessment of species composition, abundance, and relative dominance in greenhouse banana systems.

In th future, further studies could be conducted on *H. femoralis*, a species identified in Türkiye after a long absence, and for which there are extremely limited resources on its biology. Comprehensive research could be carried out on both the biological aspects of the species and its control methods (e.g., the effectiveness of chemical control methods). Furthermore, observations and research could be conducted to determine the presence of aphid species, identified for the first time on banana plants, in subsequent production seasons.

## 5. Conclusions

This study provides the most comprehensive assessment to date of the pest and natural enemy fauna associated with greenhouse banana production in the Western Mediterranean Region of Türkiye. Thirty-nine arthropod species belonging to 20 families pertaining to 8 orders were obtained from greenhouse banana cultivation during 2022–2023. The findings provide a baseline dataset on the population densities of major pests throughout the production season and document both pest and beneficial species present in banana greenhouses. Considering these data will inform the development of evidence-based, sustainable integrated pest management strategies to expand banana production areas. It is recommended that further studies be conducted to reduce pest populations and enhance the role of natural enemies.

## Figures and Tables

**Table 1:** Locations and coordinates of the periodic (15-day intervals) and nonperiodic (randomly selected) greenhouse study areas.

Periodic					
Location	Planting Date	Variety	Coordinate	Study Area (ha)	
Aksu	November 2020	Alata Azman	36.982510° N 30.899904° E	1	
Serik	July 2020	Grand Azman	36.868146° N 31.155137° E	0.8	
Manavgat	October 2020	Azman	36.863636° N 31.191507° E	0.6	
**Nonperiodic**					
**Location**	**Coordinate**	**Study area** **(ha)**	**Location**	**Coordinate**	**Study area** **(ha)**
Aksu/ Dumanlar	37.000959° N 30.902117° E	5.3	Manavgat/ Gündoğdu	36.855902° N 31.277742° E	74.35
Aksu/ Topallı	37.022655° N 30.804957° E		Manavgat/ Taşağıl	36.923388° N 31.228761° E	
Aksu/ Kurşunlu	37.059488° N 30.811408° E		Manavgat/ Denizyaka	36.863869° N 31.191381° E	
Aksu/ Solak	36.964300° N 30.901501° E		Manavgat/ Büklüce	36.874908° N 31.175037° E	
Alanya/ Toslak	36.628220° N 31.904474° E	2.6	Manavgat/ Sarılar	36.822964° N 31.429496° E	
Demre/ Küçükkum	36.240375° N 30.005847° E	10.15	Manavgat/ Ulukapı	36.815432° N 31.475678° E	
Demre/ Alakent	36.254194° N 29.987061° E		Manavgat/ Hocalar	36.891889° N 31.271537° E	
Demre/ Beymelek	36.250358° N 30.034876° E		Manavgat/ Yeniköy	36.844750° N 31.384104° E	
Demre/ Köşkerler	36.279041° N 29.999152° E		Manavgat/ Karacalar	36.746593° N 31.557175° E	
Demre/ Gökyazı	36.247966° N 29.992907° E		Manavgat/ Seydiler	36.767442° N 31.535736° E	
Gazipaşa/ Beyrebucak	36.219467° N 32.392015° E	2.75	Serik/ Kayaburnu	36.927454° N 31.015551° E	14.4
Gazipaşa/ Ekmel	36.250850° N 32.324556° E		Serik/ Karıncalı	36.958021° N 31.024749° E	
Kepez/	37.016246° N 30.772015° E	15.25	Serik/ Akocayatak	36.905565° N 30.960250° E	
Kumluca/ Beykonak	36.334751° N 30.310621° E	4.1	Serik/ Eminceler	36.902261° N 31.070099° E	
Kumluca/ Mavikent	36.310555° N 30.337671° E		Serik/ Zıpçık	36.901135° N 31.139037° E	
Kumluca/ Göksu	36.332914° N 30.268581° E		Serik/ Ykocayatak	36.953919° N 30.962720° E	
Kumluca/ Haciveliler	36.368054° N 30.272209° E		Serik/ Gebiz	37.103325° N 30.943023° E	
Manavgat/ Çakış	36.922187° N 31.186708° E		Serik/ Arahmanlar	36.977594° N 30.938514° E	

**Table 2:** Pest species detected in banana greenhouses.

Species	Order: Family
*Frankliniella intonsa* (Trybom 1895)	Thysanoptera: Thripidae
*Thrips hawaiiensis* (Morgan 1913)	Thysanoptera: Thripidae
*Thrips tabaci* Lindeman 1889	Thysanoptera: Thripidae
*Hercinothrips femoralis* (Reuter 1891)	Thysanoptera: Thripidae
*Pentalonia nigronervosa* Coquerel 1859	Hemiptera: Aphididae
*Rhopalosiphum maidis* (Fitch 1856)	Hemiptera: Aphididae
*Brachycaudus helichrysi* (Kaltenbach 1843)	Hemiptera: Aphididae
*Bemisia tabaci* (Gennadius 1889)	Hemiptera: Aleyrodidae
*Aleyrodes* sp.	Hemiptera: Aleyrodidae
*Planococcus citri* (Risso 1813)	Hemiptera: Pseudococcidae
*Dysmicoccus brevipes* (Cockerell 1893)	Hemiptera: Pseudococcidae
*Ceroplastes rusci* (L. 1758)	Hemiptera: Coccidae
*Coccus hesperidum* L. 1758	Hemiptera: Coccidae
*Chrysomphalus aonidum* (L. 1758)	Hemiptera: Diaspididae
*Tetranychus turkestani* (Ugarov & Nycolsky 1937)	Acari: Tetranychidae
*Tetranychus urticae* Koch 1835	Acari: Tetranychidae
*Spodoptera littoralis* (Boisduval 1833)	Lepidoptera: Noctuidae

**Table 3:** Natural enemies detected in banana greenhouses.

Species	Order: Family
*Neoseiulus californicus* (McGregor 1954)	Acari: Phytoseiidae
*Kampimodromus aberrans* (Oudemans 1930)	Acari: Phytoseiidae
*Scymnus levaillanti* (Mulsant 1850)	Coleoptera: Coccinellidae
*Stethorus gilvifrons* (Mulsant 1850)	Coleoptera: Coccinellidae
*Coccophagus shillongensis* Hayat and Singh 1989	Hymenoptera: Aphelinidae
*Encarsia aurantii* (Howard 1894)	Hymenoptera; Aphelinidae
*Eretmocerus mundus* Mercet 1931	Hymenoptera: Aphelinidae
*Aphidius colemani* Viereck 1912	Hymenoptera: Braconidae
*Praon abjectum* Haliday 1833	Hymenoptera: Braconidae
*Praon* sp.	Hymenoptera: Braconidae
*Aphanogmus* sp.	Hymenoptera: Ceraphronidae
*Dendrocerus* sp.	Hymenoptera: Ceraphronidae
*Asaphes vulgaris* Walker 1834	Hymenoptera: Pteromalidae
*Syrphophagus* sp.	Hymenoptera: Encrytidae
*Alloxysta citripes* (Thomson 1862)	Hymenoptera: Figitidae
*Paragus quadrifasciatus* Meigen 1822	Diptera: Syrphidae
*Lestodiplosis arcuata* (Winnertz 1853)	Diptera: Cecidomyiidae
*Lestodiplosis maculata* (Winnertz 1853)	Diptera: Cecidomyiidae
*Feltiella acarisuga* (Vallot 1827)	Diptera: Cecidomyiidae
*Scolothrips longicornis* Priesner 1926	Thysanoptera: Thripidae
*Synaphris* sp.	Araneae: Synaphridae
*Cheiracanthium* sp.	Araneae: Eutichuridae

**Table 4:** Total number, mean (± SE), and proportion of the most abundant pest families on leaves in periodically monitored greenhouses in 2022–2023 season.

Family		2022			2023			Total
		Aksu	Serik	Manavgat	Aksu	Serik	Manavgat	
Tetranychidae	n * mean ± SE (%)	190 8.6 ± 3.3 (40.3)	696 31.6 ± 7.2 (90)	889 40.4 ± 8.4 (97.1)	122 5.5 ± 2.4 (15.6)	1596 72.5 ± 10.8 (98.2)	1637 74.4 ± 12.7 (97.3)	5130
Aleyrodidae	n * mean ± SE (%)	137 6.2 ± 1.9 (29.1)	46 2.1 ± 1.0 (6)	14 0.6 ± 0.4 (1.5)	108 4.9 ± 2.5 (13.8)	14 0.6 ± 0.4 (0.9)	16 0.7 ± 0.5 (0.96)	335
Pseudococcoidae	n * mean ± SE (%)	132 6 ± 2.4 (28)	29 1.3 ± 0.8 (3.8)	0 0 ± 0 (0)	523 23.8 ± 2.4 (66.8)	15 0.7 ± 0.5 (0.9)	28 1.3 ± 0.7 (16.7)	727
Noctuidae	n * mean ± SE (%)	12 0.5 ± 0.3 (2.5)	2 0.1 ± 0.1 (0.3)	0 0 ± 0 (0)	30 1.4 ± 0.8 (3.8)	0 0 ± 0 (0)	0 0 ± 0 (0)	44
Aphididae	n * mean ± SE (%)	0 0 ± 0 (0)	0 0 ± 0 (0)	13 0.6 ± 0.4 (1.4)	0 0 ± 0 (0)	0 0 ± 0 (0)	0 0 ± 0 (0)	13
Total		471 (100)	773 (100)	916 (100)	783 (100)	1625 (100)	1681 (100)	6249

* Total number of pests.

**Table 5:** Predator number and percentage density of spider mites in 2022–2023.

Seasons	Location	Total Number of Predators on Leaf (20 cm^2^) and Abundance (%)			
		Phytoseidae	Cecidomyiidae	Coccinellidae	Thripidae
2022	Aksu	6	4	0	0
	Serik	1	2	0	7
	Manavgat	2	1	0	4
2023	Aksu	6	3	0	0
	Serik	19	2	0	6
	Manavgat	20	2	5	11
Total		54 (53.5)	14 (13.9)	5 (5)	28 (27.8)

## Data Availability

The original contributions presented in this study are included in the article. Further inquiries can be directed to the corresponding author.
